# Functional State of the Motor Centers of the Lumbar Spine after Contusion (Th8-Th9) with Application of Methylprednisolone-Copolymer at the Site of Injury

**DOI:** 10.3390/biomedicines11072026

**Published:** 2023-07-18

**Authors:** Maxim Baltin, Victoriya Smirnova, Regina Khamatnurova, Diana Sabirova, Bulat Samigullin, Oskar Sachenkov, Tatyana Baltina

**Affiliations:** 1 Research Laboratory “Mechanobiology”, Institute of Fundamental Medicine and Biology, Kazan Federal University, 420015 Kazan, Russia; baban.bog@mail.ru (M.B.); samigullinmd@gmail.com (B.S.); 2 Research Institute of Sports Reserve Training Technologies, Volga State University of Physical Culture, Sports and Tourism, Universiade Village, 35, 420010 Kazan, Russia; 3 N.I. Lobachevsky Institute of Mathematics and Mechanics, Kazan Federal University, 420008 Kazan, Russia; yaikovavictoriya@mail.ru (V.S.); sabirova.dianka@list.ru (D.S.); 4works@bk.ru (O.S.); 4 Interdisciplinary Neuroscience Faculty, Goethe-Universität Frankfurt am Main, 60323 Frankfurt am Main, Germany; hamatnurovaregi@mail.ru; 5 Department of Human and Animal Physiology, Institute of Fundamental Medicine and Biology, Kazan Federal University, 76 K. Marx St., 420015 Kazan, Russia; 6 NeuroStart Medical Center, 420049 Kazan, Russia; 7 Department Machines Science and Engineering Graphics, Tupolev Kazan National Research Technical University, 420111 Kazan, Russia

**Keywords:** methylprednisolone, amphiphilic trifunctional block copolymer, rat, motor evoked responses, gait analysis, Vicon Motion Systems

## Abstract

Spinal cord injuries must be treated as soon as possible. Studies of NASCIS protocols have questioned the use of methylprednisolone therapy. This study aimed to evaluate the effect of local delivery of methylprednisolone succinate in combination with a tri-block copolymer in rats with spinal cord injury. The experiments were conducted in accordance with the bioethical guidelines. We evaluated the state of the motor centers below the level of injury by assessing the amplitude of evoked motor responses in the hind limb muscles of rats during epidural stimulation. Kinematic analysis was performed to examine the stepping cycle in each rat. Trajectories of foot movements were plotted to determine the range of limb motion, maximum foot lift height, and lateral deviation of the foot in rats on the 21st day after spinal cord injury. We have shown that the local application of methylprednisolone succinate in combination with block copolymer leads to recovery of center excitability by 21 days after injury. In rats, they recovered weight-supported locomotion, directional control of walking, and balance. The proposed assessment method provides valuable information on gait disturbances following injury and can be utilized to evaluate the quality of therapeutic interventions.

## 1. Introduction

The lumbosacral spinal cord contains neuronal circuits that can generate locomotion of the lower limbs in various species [1,2]. Analysis of locomotor activity is important for understanding the organization and operation of spinal circuits and their downward control both under normal conditions and after motor disorders and injuries [3]. Animal models of spinal cord injury (SCI) are interesting for studying mechanisms of neural plasticity and are used quite often in the evaluation of motor function, posture, and locomotion [4,5]. In animal models, plasticity develops on its own, but it may be facilitated by various types of training [6,7,8]. However, the mechanisms responsible for changes in the spinal cord below the injury location remain unclear [9].

The SCI causes direct mechanical tissue damage (primary injury) and biochemical changes that cause progressive cell death (secondary injury) [10]. The initial injury’s severity is determined by its nature and cannot be altered. The severity of the secondary injury can be modulated by pharmacological agents, such as methylprednisolone (MP) [11].

Injection of high doses of MP was recommended based on the National Acute Spinal Cord Injury Study (NASCIS) [12] as a means of neuroprotection for 8 h after spinal cord injury. High doses of MP have shown neuroprotective efficacy in injury site reduction studies [13,14,15]. MP acts as an antioxidant and anti-inflammatory agent, which can also stabilize lysosomal membranes and suppress edema [16]. Apoptosis inhibition in oligodendrocytes is another benefit of MP [17]. Nevertheless, ongoing discussions have questioned the use of the proposed MP therapy protocol. The 2002 Guidelines for the Management of Acute SCI recommended MP for acute SCI at the discretion of the physician and patient. Despite minimal changes in the literature informing the role of an MP in the treatment of acute SCI, the 2013 Guidelines for the Management of Acute SCI recommended Tier 1 not to prescribe MP to patients with acute SCI [18].

A high dose of MP can cause several side effects, such as pneumonia, stomach ulcers, leukemia, infection, and neuropathy, with only slight improvements in neurological recovery [19,20]. It is known that the delivery of MP through systemic injection is affected by the short pharmacokinetics half-life of intravenous MP (2.5–3 h) [21] and the mediated removal of MP by P-glycoprotein from the spinal cord [22]. We hypothesize that most of the therapeutic side-effects of MP are caused by excessive doses of MP in order to maintain the concentration of MP at a sufficiently high level, given the half-life of MP. We also believe that such high doses of MF could have been prevented if MP delivery to the lesion had been targeted as precisely as possible to the area of injury. The lack of an effective mechanism for the delivery to the damaged site is probably the biggest barrier to its broad and successful clinical application. Nevertheless, MP can be delivered using biomaterials, which significantly increases its effectiveness and reduces the likelihood of side effects [20,23].

Nonionic amphiphilic polymers can reversibly change the conformation and activity of biological components such as lipid membranes, membrane transporters, and enzymes [24,25]. Polymers significantly increase the duration of drug action and allow the delivery of biologically active substances only to the target site. The most studied amphiphilic polymers include ethylene oxide (EO) and propylene oxide (PO) block polymers [26,27]. In the aqueous environment, amphiphilic triblock copolymers (TBC) can spontaneously fuse to form polymeric micelles [28,29]. The specific features of delivery systems based on nanomicelles are their small size and preservation of drug stability [30]. We have shown that the use of TBC increases the glucocorticoid concentration in the treated spinal cord by more than four times compared to other treatments [31].

In the present study, we evaluated the effects of MP in combination with TBC in the chronic and acute phases after SCI. For this purpose, we evaluated functional changes in spinal neural networks and biomechanical characteristics of locomotion in rats after SCI and subsequent MP and TBC treatments.

## 2. Materials and Methods

### 2.1. Object of Study and Bioethical Standards

All experiments were performed in accordance with ethical norms and approved by the Local Ethics Committee of the Federal State Autonomous Educational Institution of Higher Education “Kazan (Volga Region) Federal University’’ Protocol No. 2 dated 29 May 2015, and Protocol No. 30 dated 28 June 2021. Animals’ treatment prior to, during, and after experiments were performed in accordance with the principles of the Basel Declaration, the requirements of the Directive of the European Parliament and the Council of 22 September 2010 on the protection of animals used for scientific purposes and ARRIVE instructions [32,33].

All procedures were performed under combined intramuscular analgesia using zoletil (Zoletil 50, Virbac, France), 1 mg/kg, and injectable xylavet (XylaVET, Pharmamagist Ltd., Hungary) 0.05–0.10 mL/kg. Euthanasia of animals was carried out by decapitation on the guillotine.

### 2.2. Spinal Cord Injury Model

The SCI was applied at the level of the Th8 vertebra according to the modified method of A. R. Allen (1911) [34,35]. A skin incision was made directly above the site of the laminectomy, then muscle and soft tissues were moved apart with a special device. The vertebral arch and/or spinous process were then removed to allow inspection and manipulation of the spinal cord. After laminectomy (the dura mater remained intact), a 20 cm high tube was placed on the roots of the arches of the laminectomy vertebra, in which a weight of 2.5 g was lowered from a height of 5 cm.

Contraction of the lower limb muscles served as trauma control. The weight-drop impactor and the tube were immediately removed after an impact. After the surgery, the antibacterial drug Enroflon 5% (Russia) 0.1 mL/kg of animal body weight was injected to prevent infection. After injection, the animals were left to rest at a temperature of 38 °C for 2 h. In the postoperative period, in animals with impaired urination, the bladder was mechanically emptied twice a day until its function was restored.

### 2.3. Application of the Gel

After laminectomy, the decompression window was cleaned of excess blood and fluid and then dried with a sterile gauze cloth for 10 min. Then, a drop of the test gel was applied to the site of the decompression window through a 1 μL syringe until the window was completely covered with the test gel (~50 mg). Next, the open incision site was closed to prevent light from reaching the gel. Finally, the rat was placed on a heated surface for 6 h.

Bifunctional and trifunctional oxidized copolymers of ethylene oxide and propylene oxide are the new means of targeted delivery of biologically active substances to cells and tissues [26]. The triblock copolymer (TBC) was synthesized and provided by T. Abdullin, the head of the Research Laboratory of Bioactive Polymers and Peptides of Kazan Federal University.

### 2.4. Methylprednisolone Administration

Metipred 250 and lyophilizate were used to prepare a solution for intravenous and intramuscular administration (ORION CORPORATION, FINLAND). One bottle contains methylprednisolone sodium succinate (MPS) 331.5 mg, which corresponds to the content of MP—250 mg. Excipients: sodium dihydrogen phosphate dihydrate, sodium hydrogen phosphate anhydrous, and sodium hydroxide.

Intravenous administration of MPS. Animals received a single high dose of MPS (30 mg/kg) intravenously immediately after injury. Other doses were injected repeatedly 8, 24, and 48 h after the injury in the first series of experiments. In the second series of experiments, animals received a single high dose of MPS (30 mg/kg) intravenously immediately after injury.

MPS application in combination with a copolymer. Stock solutions of the copolymers were prepared in milli-Q water at a concentration of 10 mg/mL. MPS was dissolved in isotonic sodium chloride solution (0.9%) at a concentration of 62.5 mg/mL (125.9 mmol) recommended for infusion. MPS and dexamethasone (DXM) were initially dissolved in DMSO. To prepare compositions and mixed micelles consisting of glucocorticoids and copolymers, equal volumes of solutions of these two components were thoroughly mixed and left for 30 min so that the drug and copolymer combined and formed mixed micelles. Solutions were used immediately after preparation. The characteristics of the gel and its dilution have been described previously [26,31].

### 2.5. Electrode Implantation

**Figure 1:**
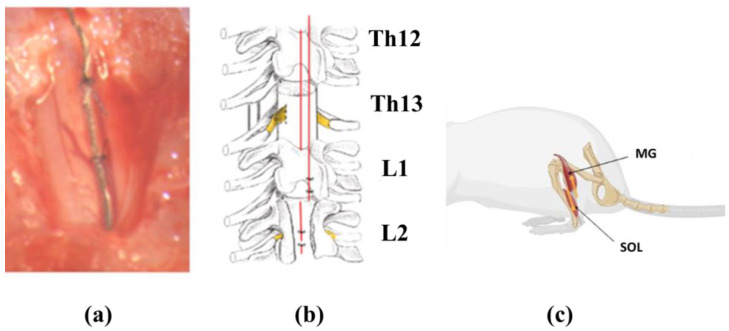
Electrode implantation: (**a**) location of the stimulation electrode on the spinal cord surface; (**b**) electrode placement diagram (indicated by red lines); (**c**) location of withdrawal electrodes in the soleus (SOL) and gastrocnemius (GM) muscles of the rat.

Teflon-coated stainless-steel wire (793500 PFA-Coated Stainless Steel, A m Systems) was passed under the spinous processes over the dura mater of the remaining vertebrae between the laminectomy sites. After removing a small portion (~1 mm) of the Teflon coating to expose the surface of the wire facing the spinal cord, the electrodes were placed in the midline of the S1 segment of the spinal cord. The electrodes were secured to the dura above and below the electrode using an ethylene surgical suture (Ethilon, 8.0). In addition, Teflon has gently pulled over the cut end of the wires to prevent stimulation through this site (Figure 1a,b).

The laterally placed wires were knotted to the midline wires above and below the electrodes to ensure the proper mediolateral spacing. The common ground wire, which had 1 cm of Teflon removed at the distal end, was secured subcutaneously in the scapular region. The wire was rolled up in the back area to provide stress relief.

Two wire electrodes were placed in each muscle (Figure 1c). An incision was made in the skin and fascia to expose the abdomen of the gastrocnemius, soleus, and tibialis anterior muscles. The wires were placed intramuscularly as previously described [36]. Muscles under study: soleus muscle (SOL)—wire electrodes were placed parallel to each other with a hook needle into the head of the muscle on the inside of the paw; gastrocnemius muscles (MG)—wire electrodes were placed at an angle to each other on the outer side of the paw.

### 2.6. Motor Function Analysis by Vicon Video Capture System

**Figure 2:**
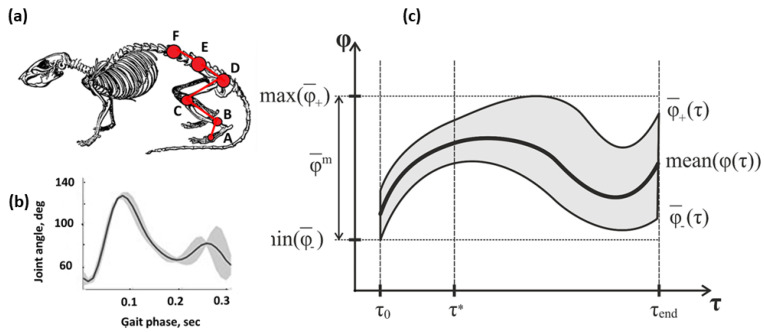
(**a**) Markers layout for movement capture: (A) metatarsal bones, (B) ankle joints, (C) knee joints, (D) tail, (E) sacrum, muscles of the back (F); (**b**)—Angulogram of the knee joint (example); (**c**)—the main characteristics calculated from the angulogram (explanation in the text).

Three-dimensional data of movement was obtained using six Vicon MX cameras (Vicon Motion Systems, Oxford, UK) placed on special mounts in a semicircle. An Active Wand calibration marker (Vicon Motion Systems, Oxford, UK) was used to calibrate and synchronize the cameras. A Sony camcorder was used to obtain a standard video. Ten passive reflective markers were placed on the shaved skin, identifying the location of the muscles of the back, sacrum, knee, ankle, and metatarsal bones (markers placement is shown in Figure 2a).

During the video recording, rats moved in an open field. Spline interpolation was used to resample the Vicon data up to 30 Hz before analysis. Gait phases were determined with time marks of gait events—foot lift-off and resumption of contact with the surface. Kinematic analysis was performed for the complete gait cycle of each rat. The frame frequency was equal to 100 Hz. The x and y axes were located in the horizontal plane; the z-axis was perpendicular to the plane. The rat movement occurred in the x-y plane.

Initial processing (model restoration and artifact filtration) was performed by the Vicon Nexus 2.5 software (Vicon Motion Systems, Oxford, UK). Further processing was performed by the author’s software in MATLAB.

The entire methodology is described in detail in [37], and features of the analysis methodology for the case of animals with SCI are disclosed in detail in [31,38,39].

Let us note the key points of motion analysis. So, the input data were as follows:

(1) $$\left\{t_{i},m_{i1}(x_{i1},y_{i1},z_{i1}),...,m_{in}(x_{in},y_{in},z_{in})\right\},\;i=\bar{1,m}$$

where *t_i_*—time step, *m_i_*—marker data, *x_i_*, *y_i_*, *z_i_*—coordinates of the corresponding marker, *m*—number of frames, and *n*—number of markers.

Then, the limb vectors can be calculated as follows:

(2) $$\left\{\begin{array}{c} \vec{DC}=\vec{D}-\vec{C}\\ \vec{CB}=\vec{B}-\vec{C}\\ \vec{BA}=\vec{A}-\vec{B} \end{array}\right.$$

where vectors *A*, *B*, and *C* are coordinates of corresponding markers (see Figure 2a).

So, angles can be calculated by equations:

(3) $$\{∠DCB=arcCos\left(\frac{\vec{DC}\cdot \vec{CB}}{\left|\vec{DC}\right|\cdot \left|\vec{CB}\right|}\right)∠CBA=arcCos\left(\frac{\vec{BA}\cdot \vec{CB}}{\left|\vec{BA}\right|\cdot \left|\vec{CB}\right|}\right)$$

To determine the steps, the local minimum of the z coordinate of point A was used. Then, for each step, the vectors *BA*, *CB*, and *DC* were calculated using Equation (1), and joint angles were calculated using Equation (2).

To analyze the change in angle, the results were averaged over all steps. Joint angle data were found for each joint. The time axis was normalized by the step phase. To estimate limb mobility after SCI, joint flexion was calculated: angle difference between values at the beginning of the swing phase and at the beginning of the push phase. To estimate movement deviations, standard deviation was calculated. Additionally, the distribution of the joint angle data was presented as follows:

(4) $${\bar{\varphi }}_{\pm }(\tau )=mean(\varphi (\tau ),N_{step})\pm std(\varphi (\tau ),N_{step})$$

where φ(τ)—joint angle, *mean*(·, *k*) and *std*(·, *k*)—mean and standard deviation of joint angle, *N_step_*—number of steps.

An example of joint angle data is shown in Figure 2c. To quantify the volume of motion, the following equation was used:

(5) $${\bar{\varphi }}^{m}=\underset{\tau }{max}({\bar{\varphi }}_{+}(\tau ))-\underset{\tau }{min}({\bar{\varphi }}_{-}(\tau ))$$

To quantify the range of motion, the area of the anatomic triangle was calculated. The vertices of the triangle where the sacrum at the beginning of the movement, the foot position at the beginning of the movement, and the maximum height of the foot. The lateral deviation of the foot was quantified as the indentation of the foot relative to the hip trajectory:

(6) $$T_{j}=\left({\vec{CB}}_{j}\times {\vec{DC}}_{j}\right)\cdot {\vec{L}}_{j}$$

where *L_j_*—the direction of movement in a step.

To quantify the ability of the foot to be lifted, the bridge of the foot was calculated by the equation:

(7) $$\underset{z}{B_{r}=\max}\left\|\vec{A}(t)\right\|-z_{0}$$

where *z*_0_—height of the floor.

For the analysis of the data, clustering was applied. For this purpose, the joint angles data were normalized by a range of angles and step time. The coefficients of polynomial interpolation of the normalized joint angle data were used for clusterization [37]. This approach allows taking into account the form of the joint angle data curve. From the biomechanical perspective, it allowed us to take into account motion patterns. For cluster visualization, the t-distributed stochastic neighbor embedding algorithm was used.

### 2.7. Electrophysiological Testing

**Figure 3:**
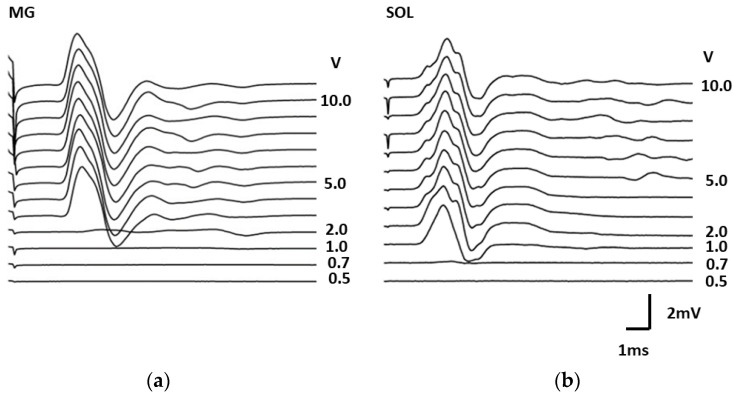
Examples of evoked motor responses were recorded in (**a**) the gastrocnemius (GM) and (**b**) soleus (SOL) muscles with a gradual increase in the intensity of electrical epidural spinal cord stimulation.

To assess spinal neural networks in the acute period of SCI, evoked muscle responses were recorded during epidural stimulation of the spinal cord. Figure 3 shows an example of the registration of responses during epidural stimulation of the gastrocnemius muscle (Figure 3a) and soleus muscle (Figure 3b).

The responses of the studied muscles were recorded before surgery, immediately after surgery, and every hour for 6 h after the contusion injury and after 3 and 21 days. Stimulation was performed using a stimulator (A m systems), and abduction, amplification, and registration of responses were performed on an amplifier (A m systems). Epidural stimulation at the L1 level was performed with single stimuli lasting 0.5 ms. Responses were recorded at stimulation intensities ranging from 0.5 to 10 V.

The maximum amplitude of evoked muscle responses was determined. The maximum peak-to-peak amplitude of each response was calculated as the average of seven responses and is presented as a percentage of the control value. Motor-evoked responses (MERs) of the gastrocnemius (MG) and soleus (SOL) muscles with a latent period of 3–6 ms were recorded in all animals during electrical epidural stimulation of the spinal cord.

### 2.8. Organization of the Experiment

**Figure 4:**
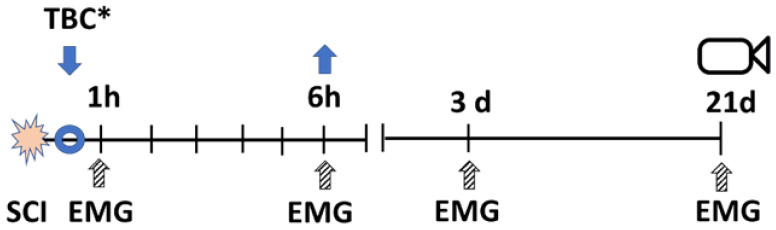
Experimental Design. The diagram shows the order of the activities in the study. SCI indicates the time of induction of the injury, followed by administering the substance. The diagram illustrates the application of TBC (down arrow). In various experimental groups, this involved injecting MPS or applying MPS with a copolymer (MPS+TBC) (*). After 6 h, the copolymer was removed (up arrow). EMG—registration of evoked muscle responses to epidural stimulation was conducted at 1, 6 h, and at 3 and 21 days after spinal cord injury (SCI); on the 21st day, the movement of the hind limbs was recorded using the Vicon system.

The overall experimental design is presented in Figure 4.

In each experimental group of animals, as shown in the diagram, a contusion injury was induced, and the tested substance was administered within the first 20 min, depending on the group. Muscle responses to epidural spinal cord stimulation were recorded in 1 h, 6 h, 3 days, and 21 days, and video analysis of animal movement was conducted on the 21st day for all experimental groups.

Rats were randomly and blindly assigned to four groups:

(1) Control group (Con; n = 7): intact animals.
(2) Untreated spinal cord injury (SCI; n = 7) group: animals underwent laminectomy and spinal cord injury.
(3) Methylprednisolone sodium succinate (MPS; n = 8) group: animals received a single high dose of MPS (30 mg/kg) intravenously immediately after injury.
(4) MPS in combination with a copolymer group (MPS + TBC; n = 8)—a complex of MPS and the polymer was applied to the dura of the injured part of the spinal cord for 6 h, after 6 h polymer was removed.
(5) Tri-block copolymer (TBC; n = 8) group: TBS was applied to the dura mater of the injured part of the spinal cord for 6 h, and after 6 h polymer was removed.

### 2.9. Data Analyses

Statistical data processing was carried out using the Original Lab program. Electrophysiological parameter data are presented as mean ± SE. Statistically significant differences were determined using ANOVA using a non-parametric modified Student’s *t*-test. The level of the criterion of statistical significance was set at *p* < 0.05.

Kinematic analysis parameter data are presented as median and lower and upper quartiles (Me (Q2; Q3)) for kinematic analysis using analysis of variance using ANOVA. Statistical significance between control and experimental groups was confirmed by the non-parametric Wilcoxon test pairwise comparisons. The significance level was set at *p* < 0.05.

## 3. Results

### 3.1. Changes in the Maximum Amplitude of the Evoked Motor Response of the Leg Muscles in Rats during Epidural Stimulation of the Spinal Cord

**Figure 5:**
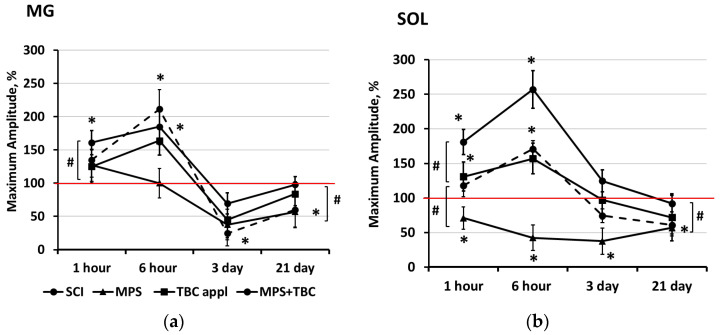
Change in the maximum amplitude of the motor evoked response to epidural stimulation of the spinal cord of (**a**) the gastrocnemius (MG) and (**b**) soleus (SOL) muscles of the rat after injury. The dotted line shows the amplitude changes in the SCI group, the triangle markers are the MPS group, the square is the group with TBC application, and the circles are the group with MPS + TBC application; The maximum amplitude is presented as a percentage of the preoperative values indicated by the red line; significance compared with preoperative values—* *p* < 0.05, between groups—# *p* < 0.05.

The maximum amplitude of the evoked response of the soleus (SOL) and gastrocnemius (MG) muscles after injury increased to 6 h compared with the values before the contusion (Figure 5). The maximum increase was noted for MG in the MPS + TBC group; the amplitude was 211 ± 29% (*p* < 0.05). In the soleus muscle, the amplitude of the response decreased in the group with intravenous MPS; after 6 h, the amplitude was 42 ± 18% (*p* < 0.05). 

Three days after SCI, a decrease in the maximum amplitude of the evoked response of the rat leg muscles was observed in all groups (Figure 5). 

Thus, in MG (Figure 5a), the maximum drop was noted in the group in the SCI, where the amplitude was 24 ± 29% (*p* < 0.05), in Sol (Figure 5b), with the intravenous MPS group, where the amplitude was 37 ± 19% (*p* < 0.05). In animals in the MPS + TBC application group, a large amplitude of the evoked response remained—124 ± 16% (*p* < 0.05) Sol and 70 ± 36% (*p* < 0.05) MG, respectively. By day 21 after the injury, the amplitude of the evoked muscle responses was restored. However, in the MPS+TBC group, the amplitude recovered to the control values, and in the groups with SCI and MPS, it was lower than before the contusion (see Figure 5).

### 3.2. Motion Estimation Using Vicon

**Figure 6:**
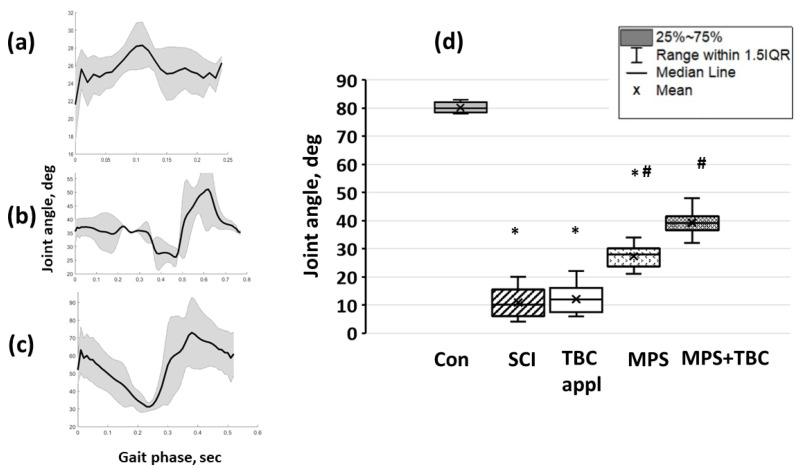
Knee flexion angle in animals of different experimental groups: (**a**–**c**)—angulograms of the knee joint in groups of animals with SCI, MPS, MPS+TBC; (**d**)—maximum knee flexion angle in animals of different experimental groups: in control (Con), with an injury of a spinal cord (SCI), with an injury of a spinal cord and applique of copolymer (TBC appl), with an injury of a spinal cord and intravenous infusion of methylprednisolone sodium succinate (MPS), with an injury of a spinal cord and local delivery of methylprednisolone sodium succinate in a complex with copolymer (MPS+TBC); *—*p* < 0.05—significance relative to the control group; #—*p* < 0.05—significance of results relative to the group with contusion injury.

Consider knee joint angles for intact animals. Changes in the first third were from 43° ± 2° to 123° ± 5°, in the second third were equal to 67° ± 2° and in the third returned to the values of the first third. In the SCI group, the rats were unable to consistently repeat the step cycle at a constant frequency, walking was interrupted, and the foot was dragged along the support; however, separate biphasic steps were present. In the first third of the cycle, the push (swing) phase ended, and inadequate movements were observed with the initiation of movement in the knee. So, the rat squeezed its hind paw, and then a low-amplitude push was followed. The knee joint angle changed from 25° ± 2° to 28° ± 3°. In the swing phase angle changed from 27° ± 3° to 25° ± 3° but then was equal to 24° ± 2° on average (see Figure 6a). In the TBC group, there was the same motion behavior. Motion activity in MPS and MPS+TBC groups increased (see Figure 6b,c). Flexion in the knee joint in the MPS group was 38° ± 6° (*p* ≤ 0.05) in the MPS+TBC group, and the angle at the knee joint changed from 32° ± 4° (with SCI) to 70° ± 10° (*p* ≤ 0.05).

An average knee joint angle of an intact animal was equal to 80 (75.8; 82)°. In the SCI group; after 21 days, the average knee joint angle was 10 (6; 15.5)° (*p* < 0.05). In MPS and MPS + TBC groups, an average knee joint angle increased up to 28° (23.5; 30)° and 39 (36.5; 41.5)° (*p* < 0.05), respectively (see Figure 6d).

**Figure 7:**
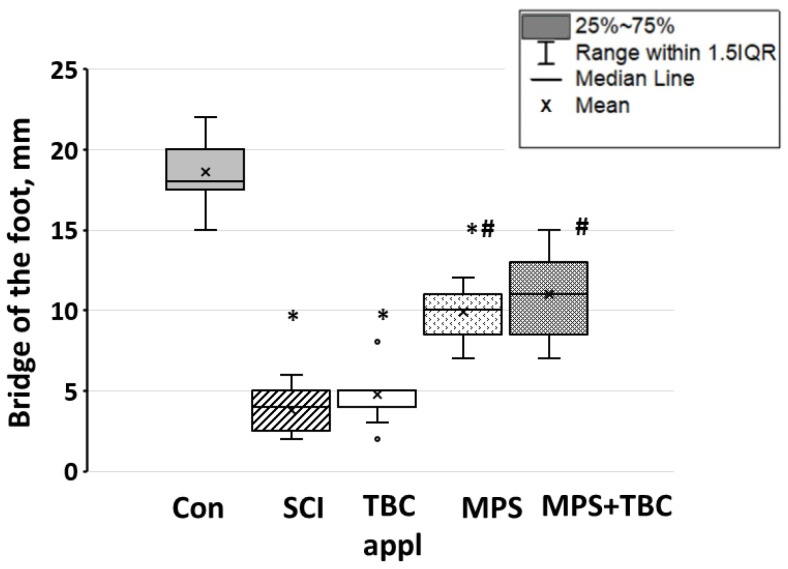
Maximum bridge of the foot in experimental groups of animals: designations as in Figure 6; *—*p* < 0.05—significance relative to the control group; #—*p* < 0.05—significance of results relative to the group with contusion injury.

In the control group, the animals lifted their foot by 18 (17.5; 20) mm (see Figure 7, labeled “bridge of the foot”). In the SCI group bridge of the foot decreased. In addition, the rats in the SCI group were unable to reproduce an adequate step cycle. Figure 7 shows that the maximum bridge of the foot in the SCI group was 4 (2.5; 5) mm (*p* < 0.05). This indicates a violation of the movements of the rat’s hind limbs after SCI. The injury led to the impossibility of developing sufficient muscle strength to raise the foot and maintain its own weight. In the MPS + TBC groups bridge of the foot close to the control group and was equal to 11 (8.5; 13) mm.

**Figure 8:**
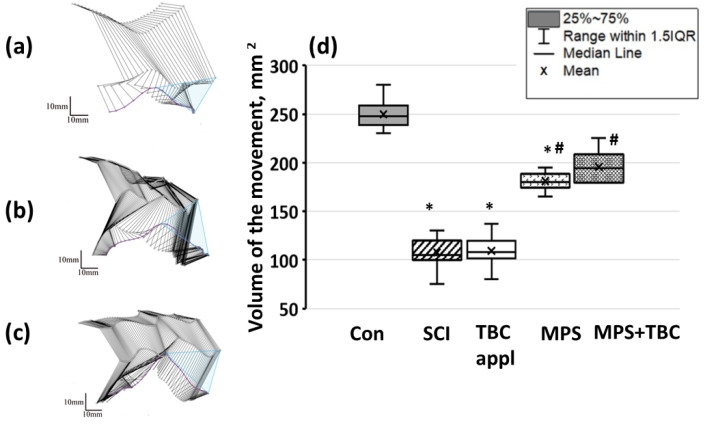
A representative image of the position of the hind limb of the rat in the step phase (**a**–**c**): the purple line is the trajectory of the foot, the blue triangle is the volume of movement of the hind limb; (**a**)—group SCI; (**b**)—group MPS; (**c**)—group MPS + TBC; (**d**) volume of movement of the leg in experimental animals: designations as in Figure 6; *—*p* < 0.05—reliability relative to the control group. #—*p* < 0.05—reliability of results relative to the group with a concussion injury.

The hind limb range of motion in the Con group was 248 (238.5; 258.5) mm^2^ (Figure 8). In the SCI and TBC groups, the hind limb range of motion was 105 (99.5; 120) mm^2^ (*p* < 0.05), see Figure 8. It should be noticed that the group’s rats were unable to reproduce the gait cycle successively with a constant frequency. Abnormal gait was characterized by foot-dragging along support, but both gait phases were presented. In the MPS and MPS+TBC groups, gait quality improvement was observed. So, the hind limb range of motion in the groups was 180 (174; 188) mm^2^ (*p* < 0.05) and 194 (179; 208) mm^2^ (*p* < 0.05), respectively.

**Figure 9:**
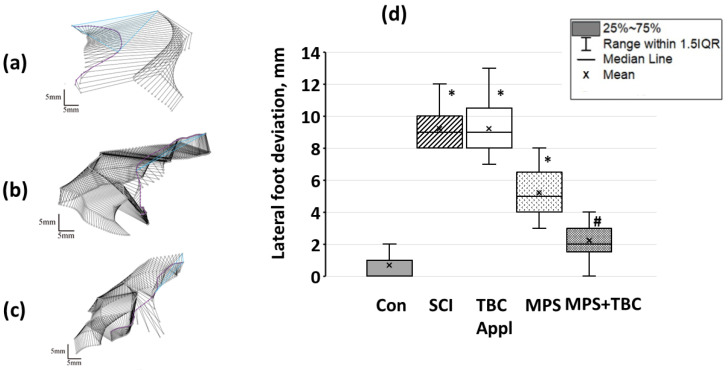
Representative image of rat hind limb movements in three-dimensional projection in the SCI (**a**), MPS (**b**), and MPS + TBC (**c**) groups. The blue line marks the trajectory of the rat body (sacral area) movement, and the blue triangle marks the lateral deviation of the foot. (**d**) Lateral foot deviation in experimental groups of animals: designations as in Figure 6; *—*p* < 0.05—reliability relative to the control group. #—*p* < 0.05—reliability of results relative to the group with a concussion injury.

Proceeding to lateral foot deviation analysis, it should be pointed to the control group, where the smallest values were observed (0.66 (0; 1) mm, see Figure 9). The rats were able to confidently rest on the foot surface and maintain their own weight. In the SCI group, rats almost did not move the hind limbs, and lateral foot deviation was 9 (8; 10) mm.

In the TBC group, rats were not able to reproduce movement, and the hind limb remained bent throughout the locomotor. The lateral deviation was 9 (8; 10,5) mm (*p* < 0.05). In the MPS group, the lateral deviation was 5 (4; 6.5) mm. In the MPS +TBC group, the lateral value was 2.0 (1.5; 3) mm (*p* < 0.05). The rats were able to rest on the foot surface and maintain their own weight.

Thus, in terms of lateral foot deviation, the group treated with MPS + TBC showed better recovery of movement function. Additionally, these emphasize a fact of a positive effect on the restoration of rat hind limb movements in the case of SCI.

**Figure 10:**
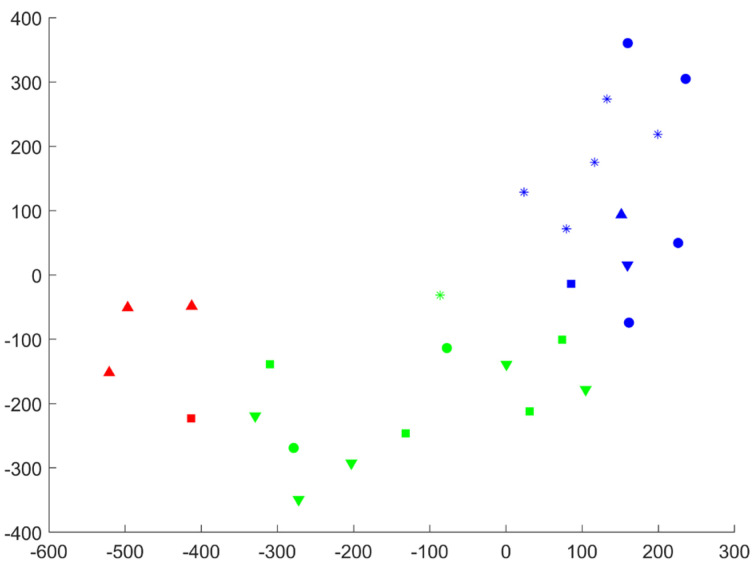
Received clusters: red—1st cluster, green—2nd cluster, blue—3d cluster; asterisk—Con group, downward-pointing triangle—SCI group, square—MPS group, upward pointing triangle—MPS + TBC group, circle—TBC group.

Focusing on the clustering results, it could be mentioned that the MPS+TBC group (red upward-pointing triangles in Figure 10) forms the 1st cluster group. 

There are some deviations (red square) that can be associated with the sample size. The 2nd cluster group majorly consists of the MPS group and the SCI group (green squares and downward-pointing triangles, respectively, in Figure 10). The 3rd cluster group majorly consists of the Con group and the TBC group (blue asterisks and circles, respectively, in Figure 10), but the maximum number of outliers appears here.

Such results suggest that the movement pattern in the MPS + TBC group has significant differences compared to the other groups. On the other hand, the second cluster shows that the movement pattern in the MPS and SCI groups is similar. This shows that MPS alone has no significant effect on the recovery of movement function. The 3rd cluster gives ambiguous results because it consists of Con and TBC groups. Some data in the 3rd cluster can be understood as outliers because they are placed on the border of the cluster. Additionally, it can be noted that some data from MPS, TBC, and SCI groups are placed on the common border of the 2nd and 3rd clusters. Thus, accessory to a certain cluster can be questioned. Moreover, the point cloud for the Con group is differentiated in this cluster, so we assume that more samples will lead to the 4th cluster. 

## 4. Discussion

Regardless of the severity of SCI, nerve tissue damage is irreversible and currently not curable. Acute therapeutic interventions are mostly limited to spinal stabilization and prevention of further damage to the brain tissue [40]. The SCI in the acute phase results in the initiation of a delayed, progressive and self-propagating wave of tissue destruction. The second phase, following the acute SCI phase, is often referred to as “secondary trauma”. This phase is characterized by the formation of a glial scar and irreversible loss of distal axonal segments due to Wallerian degeneration. Loss of the white matter of the spinal cord, which is not replaceable, causes loss of locomotion, which accompanies the chronic phase of injury [41]. 

Neuroprotection, one of the most important strategies for the recovery of damaged neural tissue, is necessary in the acute phase of SCI. The goal is to minimize and/or prevent the spread of secondary spinal cord damage, thereby reducing cellular apoptosis or necrosis and contributing to neuronal and axonal survival [42]. The search for a treatment that could reduce the neurological damage resulting from secondary spinal cord injury is ongoing.

One of the standard treatments for acute spinal cord injury is the administration of large amounts of the steroid MP within hours after injury, although the efficacy and safety of this therapy are currently in question [43]. Recently published guidelines state that MPS should not be prescribed [44,45].

The protocol for the clinical use of MP is based on the results of three large randomized clinical trials of the National Acute Spinal Cord Injury Study (NASCIS). The second of these NASCIS studies had the greatest impact on the use of MP in clinical practice [46]; the use of this particular method was the most cited in the treatment of SCI using MP [47].

Nevertheless, intravenous administration of high doses of MP in acute SCI has become controversial because of the risk of potential side effects and the small positive effect [48,49]. It has also been shown that high doses of MP do not promote the proliferation of nerve cells [50] and cannot lead to a significant improvement in neurological recovery in model animals with SCI [51]. 

However, physicians who support the use of MP in acute SCI believe that SCI is a serious enough disease to merit treatment despite the risk of serious complications. This is comparable to the use of chemotherapeutic agents in malignant diseases. This is particularly important as SCI patients place great importance on restoring their motor function, even when improvements are small [52]. Thus, it was reasonable to assume that since most of the side effects of MP therapy were closely related to the high systemic dose of MP, which led to further health complications, another way of administering the substance was needed that would address these problems.

A drug delivery system can effectively improve the therapeutic effect of drugs, reduce drug toxicity, and improve patient dependence on drugs. Ideally, such a system should have good biocompatibility and protect the drug, avoiding degradation while maintaining its stability and activity [25]. Hydrogels are widely used as neural repair materials, and in recent years, relatively good effects of hydrogels in the treatment of SCI have been demonstrated. Hydrogels can encapsulate drugs or carriers containing drugs for sustained local release [53,54].

Among polymeric carriers, the nonionic triblock copolymer Poly (ethylene oxide-poly and propylene oxide) was found to be nonimmunogenic and capable of inhibiting P-glycoprotein activity [55,56,57]. In the research laboratory, “Bioactive polymers and peptides”, KFU (supervisor T. I. Abdullin, Ph.D.) obtained a new means of delivery of biologically active substances in cells and tissues of the body, which is an oxidized modification of trifunctional oxidized copolymers of ethylene oxide and propylene oxide with carboxyl group. The results showed that the modification of the copolymers significantly improved the characteristics of the polymeric micelles. It was demonstrated that the oxidized copolymers facilitated local delivery to intact spinal cord tissue and promoted fluorescent probe accumulation in nerve tissue cells [26]. It was also shown that the complex of TBC and MPS is able to undergo self-assembly to obtain uniform and relatively stable micelles, which are characterized by high encapsulation efficiency and cellular availability of MPS. The initial study of the preparation demonstrated increased cellular uptake and antiradical activity of the complex compared to free MPS [29]. These data confirmed the possible use of TBC/MPS as a pharmaceutical composition.

Previously, we conducted a study to analyze the effects of MPS and the complex of MPS with block copolymer on the muscular system and locomotion of the grape snail [58]. It was shown that the MPS solution, getting into the hemolymph of the animal, already 1 h after administration, slows down the speed of locomotion of the snail, reducing the contractile activity of the clam soleus muscle. At the same time, the complex of MPS with block copolymer prevents this effect during the first two days of the administration, and the negative effect on locomotion begins only two days after the start of the administration, and the decrease in locomotion, in this case, is not accompanied by a decrease in the contractile activity of the muscle [58]. In a rat contusion injury model study, we have shown that there are potential advantages of local MPS delivery with TBC over conventional systemic MPS delivery in the acute phase of SCI—an improved therapeutic effect due to prolonged and targeted delivery of MPS to the injured spinal cord [31] and that local delivery of MPS in combination with TBC helps to reduce the volume of white and gray matter lesions in the spinal cord below the injury site [59]. One of the most devastating consequences of SCI is the impairment of the ability to perform locomotion. In the present study, we evaluated the effects of MPS in combination with copolymer on motor function in rats after thoracic spinal cord contusion injury. 

Much effort has gone into developing methods to analyze the movement of rats after experimental SCI. Currently, the 21-point Basso, Beatty, and Bresnahan musculoskeletal recovery scale (BBB-scale) [60] is the most widely used open-field test, and it is a reliable way of assessing motor function after thoracic SCI. At the same time, BBB-scale and similar measurements take into account several basic motor characteristics while lacking sensitivity to locomotor variability [61]. Previously in our studies, we saw no change in BBB-scale using local intraoperative hypothermia and MPS in rats with spinal cord contusion injury [62]. With the development of computer technology, the use of instrumental analysis of rat locomotion for research has become very common [63]. We use the original method of movement estimation to characterize locomotion and provide a reliable measure of the change and recovery of rats’ motion as presented in the methods. 

To assess the therapeutic effects of MPS + TBC, we conducted an electrophysiological evaluation of the motor centers of the spinal cord. One hour after injury, there were almost no changes in the parameters of the evoked tibial muscle responses to epidural stimulation. After 6 h, there was an increase in the amplitude of the mean component of the evoked muscle response, which may indicate increased excitability of the motor centers of the spinal cord. With MPS treatment, there was a decrease in the amplitude of the evoked responses and an increase in the latent period. However, the use of MPS in combination with the copolymer had the opposite effect. We observed a drastic increase in the amplitude of the evoked muscle response. By 21 days after SCI, the parameters of the evoked responses of the rats’ hind limb muscles had practically recovered to the control values. 

The observed excitation of the spinal cord centers is known to be caused by the elimination of descending supraspinal inhibitory inputs from the rostral segments of the spinal cord and from the brain stem [64]. This mechanism is thought to be a common mechanism of hyperexcitation during the acute (several hours) and chronic (several weeks) phases after SCI in rats. It is also suggested that changes in electrophysiology after SCI may be related to the loss of afferent information from paralyzed muscles (so-called homeostatic plasticity, as discussed in [65]). SCI triggers structural plasticity in the spinal cord circuit, spontaneous extensive remodeling based on the formation and removal of axonal processes, and changes in dendritic morphology [66,67,68,69]. These structural changes can affect synaptic integration and lead to changes in the electrophysiological properties of neurons [70,71]. 

Following experimental SCI in rodents, changes in the spinal cord circuit include an increase in excitatory postsynaptic potentials (EPSP) of Ia-group afferents, changes in H-reflexes, and changes in resting potential in motoneurons [71,72]. Despite the potential for recovery, strong evidence suggests that an imbalance of excitatory and inhibitory neurotransmitters after SCI in and around the epicenter of the lesion can lead to functional inactivation of the preserved tissue [73]. It can be assumed that the MPS+TBC complex can regulate activated pro-inflammatory microglia to reduce secondary damage and improve the microenvironment at the injury site, thereby promoting axonal regeneration and maintaining the overall excitability of spinal networks below the lesion site. However, there is still no information as to whether inhibition or enhancement of the excitability of surviving spinal neurons would be beneficial for functional recovery after SCI. Therefore, we performed a kinematic evaluation of movement in injured rats.

The kinematics of movement on a flat surface are highly stereotypical. Gravity is the main limiting factor, so the efficiency of movement is directly dependent on gravity, creating force to support the body and movement during the support phase of the gait cycle. Performing out the swing phase is necessary for continued movement, proper paw replacement, and to gain body acceleration. Rats walking on a plane surface distribute their body weight evenly on the forelimbs and hind limbs, providing a braking motion mainly with the forelimbs and movement mainly with the hind limbs [74]. SCI changes this kinematic pattern, the localization, and the degree of impairment, by which both the severity of the impairment and the effects of compensation can be determined. 

Our results showed that there is a significant difference in stepping motion between the SCI and MPS groups, with improvement in motor function of the MPS group animals. This indicates that MP may attenuate the effects of secondary spinal cord injury, leading to improved motor function, possibly, as shown in the literature, through an anti-inflammatory effect [75,76,77]. We did not find any evidence of significant improvement in motor function in the TBC group compared to the SCI group, but cluster analysis showed that this group may be close in pacing parameters to the control group. It is shown that copolymers may have a neuroprotective effect; it is noted that perhaps the interaction of polymers causes two separate parts of the membrane to bind to each other, effectively closing off sites of cellular damage and stopping the invasion of unwanted ions and molecules [78,79]. The application of TBCs and the MPS + TBC complex may have led to changes in conduction in the injured spinal cord segment, as manifested by changes in the excitability of lumbar thickening neurons. When analyzing the recovery of motor function, we observed an improvement in functional deficit in the MPS + TBC group, indicating that the use of local application of the MPS + TBC complex promotes a better recovery of motor function in animals that have suffered spinal cord injury. 

Motor function is controlled by several pathways, an adaptive network of descending pathways with overlapping functions and spinal networks, including reflex pathways, short and long propriospinal pathways, and pattern-generating circuits (rhythmic) [71,80]. This provides a high degree of compensation based on the redistribution of neuronal circuits after neural damage, which contributes significantly to the non-linearity of recovery. Moreover, it has been shown that in rats, pelvic limb pacing can be mediated exclusively through propriospinal networks caudal to the spinal cord lesion [81], i.e., standing, stepping, and general locomotion in SCI models are controlled locally and generated in the spinal cord and thus are less dependent on supraspinal control. Two different types of pelvic limb position coordination after SCI can be identified: coordination in the sagittal plane (mediated by propriospinal pathways) and pelvic limb position in relation to the trunk in the lateral plane (dependent on supraspinal influences). Our results showed that the use of MPS, both intravenously and in combination with a polymer, promotes the restoration of stepping movements in the sagittal plane, that is, the activation of propriospinal pathways. Additionally, lateral stability, i.e., the ability to place the feet in the correct position in the lateral plane relative to the body, was better expressed in the MPS + TBC group, indicating a more advanced stage of recovery, which depends on the connections between the trunk and the spinal cord [82].

Study limitations. The use of moderate spinal cord contusion injury determines the possible variability of functional recovery in rats [83]. We also did not use the toe segment in the analysis. However, although rats use the toe gait, most other studies also exclude this segment from rat gait. The reason for this is probably due to the small size and independent movement of the toes, which reduces the ability to accurately track the segments.

## 5. Conclusions

We have demonstrated that the local administration of MPS, together with TBC, has a facilitating effect on the spinal cord’s motor centers within the first six hours and results in the restoration of the centers’ excitability by day 21 following SCI. MPS application, in combination with copolymer, provides maintenance of excitability of the spinal centers of the lumbar spinal cord, which may serve as a basis for better motor function recovery. 

Using a novel method for processing motion video analysis data, we demonstrated the restoration of motor functions in rats. We have shown that quantification of rat pelvic limb movements in two planes (sagittal and lateral) allows measurement and a more accurate interpretation of the effects of therapeutic interventions. Our results demonstrated that an extended analysis using both dynamic gait analysis and joint kinematics proved to be a reliable method for quantifying the gait of rats with SCI and groups with different treatments.

## Data Availability

No data available.
